# Dilated convolution network with edge fusion block and directional feature maps for cardiac MRI segmentation

**DOI:** 10.3389/fphys.2023.1027076

**Published:** 2023-01-26

**Authors:** Zhensen Chen, Jieyun Bai, Yaosheng Lu

**Affiliations:** ^1^ Guangdong Provincial Key Laboratory of Traditional Chinese Medicine Information Technology, Jinan University, Guangzhou, China; ^2^ College of Information Science and Technology, Jinan University, Guangzhou, China

**Keywords:** automatic segmentation method, cardiac MRI, dilated convolution, medical image processing, deep learning

## Abstract

Cardiac magnetic resonance imaging (MRI) segmentation task refers to the accurate segmentation of ventricle and myocardium, which is a prerequisite for evaluating the soundness of cardiac function. With the development of deep learning in medical imaging, more and more heart segmentation methods based on deep learning have been proposed. Due to the fuzzy boundary and uneven intensity distribution of cardiac MRI, some existing methods do not make full use of multi-scale characteristic information and have the problem of ambiguity between classes. In this paper, we propose a dilated convolution network with edge fusion block and directional feature maps for cardiac MRI segmentation. The network uses feature fusion module to preserve boundary information, and adopts the direction field module to obtain the feature maps to improve the original segmentation features. Firstly, multi-scale feature information is obtained and fused through dilated convolutional layers of different scales while downsampling. Secondly, in the decoding stage, the edge fusion block integrates the edge features into the side output of the encoder and concatenates them with the upsampled features. Finally, the concatenated features utilize the direction field to improve the original segmentation features and generate the final result. Our propose method conducts comprehensive comparative experiments on the automated cardiac diagnosis challenge (ACDC) and myocardial pathological segmentation (MyoPS) datasets. The results show that the proposed cardiac MRI segmentation method has better performance compared to other existing methods.

## 1 Introduction

Cardiovascular disease has been widely concerned by the medical community because of its harmfulness Cai et al. (2015). With the development of cardiac imaging technology, medical staff have been able to further study this disease. Among them, short-axis cardiac magnetic resonance imaging (MRI) is adopted by medical staff due to its non-invasive imaging characteristics, and is often used for the diagnosis of cardiovascular diseases Ripley et al. (2016). In clinical cardiology, clinicians need to distinguish left ventricle (LV), right ventricle (RV), and myocardium (MYO) from short-axis cardiac MRI. Manually identifying the parts of the heart is time-consuming, tedious and susceptible to external influences. Therefore, a great method that can automatically perform cardiac MRI segmentation task is very necessary. It allows an inexperienced person to easily complete the segmentation job.

In recent years, with the development of deep convolutional networks (CNNs), many natural image segmentation (Cheng and Li, 2021; Aganj and Fischl, 2021) and medical image segmentation (Pang et al. 2021; Oksuz et al. 2020) methods have been proposed in the field of computer vision and achieved great success. U-Net Ronneberger et al. (2015) is one of the seminal works in medical image segmentation task. It has been demonstrated that segmentation of cardiac MRI with deep neural network is better than other traditional computer vision and machine learning methods Bernard et al. (2018). After U-Net was proposed, many works were improved based on u-shaped network. Most of the best performing ventricular segmentation algorithms can be roughly divided into 2D methods and 3D methods. 2D methods take a single 2D slice as input, while 3D methods utilize entire volumes. NnU-Net Isensee et al. (2019) adopts two different fusion strategies of 2D and 3D to obtain the best model. Subsequently, Ke et al. (2018) propose a method that utilizes the optimal neighborhood size of each semantic class to optimize the adversarial loss in various situations. Dangi et al. (2019) propose a network that could predict the uncertainties associated with semantic segmentation and pixel-level distance graph regression, and the loss of the network is weighted by the reciprocal of the corresponding uncertainties. Painchaud et al. (2019) propose an adversarial variational autoencoder that can be adapted to any heart segmentation method. The encoder can automatically bend an inaccurate heart shape to a close but correct shape. Oksuz et al. (2020) propose a network that could automatically correct motion-related artifacts, and the network achieved good image quality and high segmentation accuracy in the presence of synthetic motion. Yang et al. (2021) propose a deep dilated block adversarial network, which uses the properties of dilated convolution to acquire and connect multi-scale features.

However, there is still room for improvement in existing methods. The existing networks (Ronneberger et al., 2015; Cheng et al., 2020) usually use ordinary convolutional networks. In this way, it is easy to lose information or add too much information so that the features can not be fully utilized. Some methods (Dangi et al., 2019; Painchaud et al., 2019) do not take into account the fuzziness and inhomogeneity of MRI artifacts, which can easily lead to the problem of blurring between classes and unclear boundaries. In addition, some models (Isensee et al., 2019; Zhou et al., 2021) require high memory and computational costs, making their usefulness limited.

In order to solve the problem that feature information cannot be fully utilized due to the loss of effective information or the increase of invalid information, we propose a dilated convolutional network with directional feature mapping inspired by Wang et al. (2018); Cheng et al. (2020). The network is based on the U-Net architecture, which we call DDFN. In DDFN, a dilated convolution module processes the characteristics of each layer of input in the U-Net encoder and decoder. The dilated convolution module consists of three dilated convolution with different dilated rates. Note that the dilated convolution module does not change the feature size. The dilated convolution block can extract multi-scale features effectively, and it is not easy to cause feature information loss. In the decoder, the features of each layer are up-sampled to the size of the original image and then concatenated to make full use of the feature information at different stages. In addition, we propose an edge fusion block (EFB) to preserve the image boundary. In the decoding phase, EFB integrates the edge feature into the side output feature of the encoding layer. Then it is concatenated with the upsampled features in the decoding layer. Finally, we add a direction field module before the output layer of U-Net. This module uses the learned direction field to improve the original segmentation features and serves as the input to the final output module to get the final segmentation result. Experimental results show that our proposed model is more competitive than other models.

The main contributions of this paper are as follows.

1) We propose a deep learning-based cardiac MRI segmentation network. The network can effectively extract and utilize multi-scale information, and is not easy to cause loss of feature information or increase of useless information.
2) We propose an edge fusion block to integrate edge feature maps into U-Net. The purpose is to preserve more boundary information for better cardiac MRI segmentation.
3) The network combines the direction field module to enhance the differences between classes and the similarity within classes. This module uses directional feature to improve the original network features and generate the final segmentation results.

The rest of the article follows. Section 2 describes the related work. In Section 3, we describe the proposed network structure in detail. The experimental results are presented and analyzed in section 4. Finally, the conclusion is drawn in Section 5 and future work is discussed.

## 2 Related work

In this section, we will outline the related efforts from three aspects.

### 2.1 Development of medical image segmentation

Since 2000, some researchers have been trying to use computers to automatically divide different parts of the heart. Therefore, the cardiac segmentation method based on machine learning came into being. For example, Codella et al. (2008) propose a semi-automatic segmentation method to segment LV, which utilizes region growing to improve performance. In order to overcome the influence of nipple muscle on segmentation effect, Pluempitiwiriyawej et al. (2005) propose a new stochastic active contour scheme. Zhang et al. (2020) propose a new external gradient vector manifold flow over manifold. Subsequently, some scholars propose to use prior probabilistic atlas to obtain more efficient models Mitchell et al. (2001); Lorenzo-Valdés et al. (2004). The model can achieve good performance under the premise of sufficient prior knowledge. Machine learning methods have certain shortcomings, such as the need for human assistance and the difficulty of improving accuracy.

With the development of deep learning in the field of computer vision, some scholars have proposed many automatic segmentation methods based on deep learning. Shelhamer et al. (2016) propose the full convolutional machine network (FCN), which has had a profound impact on the task of semantic segmentation. For medical image segmentation task, Ronneberger et al. (2015) propose U-Net. U-Net is also a fully convolutional network, which solves the problem of small amount of medical image data. It learns feature content better by connecting features of the same size. Subsequently, for small training sets, Ngo et al. (2017) propose to combine deep learning and level sets to solve the problem. Wang W. et al. (2019) use a subdivision component and a regression component to solve the problem caused by different ventricular heights in heart segmentation. Uslu et al. (2022) propose a multi-task network to generate left atrial segmentation image and edge mask simultaneously. The network can segment edge pixels well. In the unsupervised field, Vesal et al. (2021) propose a new multi-modal MRI segmentation model based on unsupervised domain adaptation. This party can adapt network characteristics between source target domains. Wu and Zhuang (2021) designed two networks based on variational autoencoders and regularized them to reduce the difference between segmentation results and ground truth.

All the above methods are based on deep learning, which proves that deep learning can further improve the segmentation performance.

### 2.2 Dilated convolution

Holschneider et al. (1990) first propose the concept of dilated convolution and applied it to wavelet decomposition. Dilated convolution is to insert different distances between the pixels of the ordinary convolution kernel to enlarge the receptive field of the convolution layer. Dilated convolution can effectively extract features in deep learning without increasing the number of parameters. Yu and Koltun (2015) propose to introduce dilated convolutions into the model to aggregate feature information at multiple scales. Chen et al. (2017) propose a spatial pyramid pooling module to obtain multi-scale feature information through dilated convolutions of different rates in parallel. Dilated convolutions can also be applied to computer vision fields such as object tracking Hsu and Chen (2022), audio generation Oord et al. (2016), and image super-resolution Song et al. (2022).

### 2.3 Directional feature

In addition, some scholars try to improve the semantic segmentation model by using directional information. Wang Y. et al. (2019) propose a model that could learn image context information, which can explicitly encode the relative positions of semantically meaningful entities to better deal with large object portions. Xu et al. (2019) propose a new text detector for irregular scene text detection, which uses a full convolutional network to learn the direction field from the nearest text boundary to each text point. However, semantic segmentation methods for natural images often produce inaccurate results for cardiac MRI segmentation tasks. Therefore, it cannot be directly used in the field of cardiac MRI segmentation. Influenced by Cheng et al. (2020), we use the directional information to improve segmentation features to improve the performance of the model.

## 3 Proposed method

In this section, we will detail the structure of our model.

### 3.1 Network architecture

**FIGURE 1:**
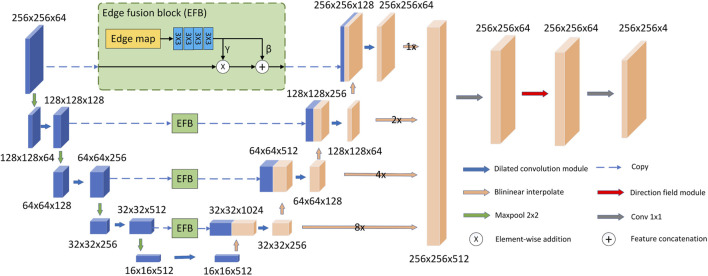
Overall structure of the proposed DDFN model.

As shown in Figure 1, our proposed model follows the U-Net model architecture. The model consists of an encoder, a decoder, EFBs and a directional field module. First, in the decoder and encoder, we replace the two consecutive 3 × 3 convolutional layers in the original U-Net with a more efficient dilated convolutional module. The purpose is to use dilated convolution to obtain larger receptive field and multi-scale feature information. In addition, we propose EFB to preserve image boundaries. In the decoding stage, EFB embeds the edge features into the downsampled features of the same size as the upsampled features, and concatenates them with the upsampled features. Second, the model upsamples the feature size of each layer of the decoder to the same size as the original image size. They are then concatenated and fused through a 1 × 1 convolutional layer. Final, the fused features are used as the input of the directional field module. The model uses the directional field to refine the fused features and generate the final segmentation result. The output segmentation map has four channels representing the probabilities of LV, RV, MYO and background.

### 3.2 Dilated convolutional module

Blurred shadows are created during MRI acquisitions due to the beating of the heart. To solve this problem effectively, we use dilated convolution to obtain multi-scale features. This reduces the impact of blurry shadows and increases prediction accuracy. Because different receptive fields can obtain different scale features, multi-scale features can reduce the error caused by heart beating. However, if large convolution kernels are used to learn large-scale features, the computational cost and number of parameters will increase significantly. In limited data sets, this situation can easily lead to overfitting. Therefore, by using dilated convolution, the acceptance domain can be extended without adding too many parameters and computing costs. Therefore, we ended up choosing dilated convolutions. The definition of dilated convolution is as follows:

$$D\left(p\right)=\underset{s+lt=p}{\sum }F\left(s\right)k\left(t\right),$$ (1)

where $F:Z^{2}\to R$ represents the input of the dilated convolution. The convolution kernel of size (2*r* + 1)^2^ is represented by $\Omega_{r}={\left[-r,r\right]}^{2}\cap Z^{2}$ and $k:\Omega_{r}\to R$ . *D*(⋅) represents the output of the convolution operation, where *l* represents the dilation rate, *s* is the stride, and *p* is an element of *D*(⋅).

**FIGURE 2:**
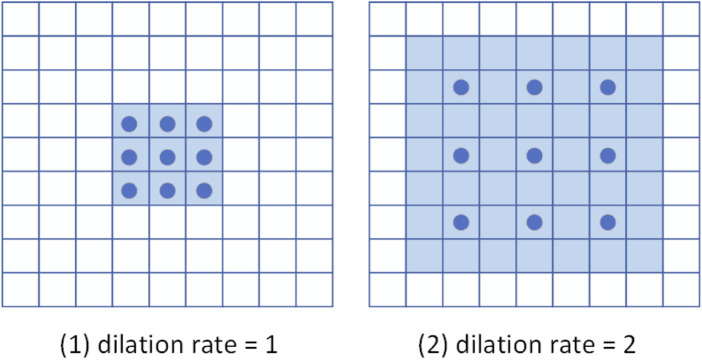
Schematic diagram of dilated convolution. The dark blue points represent the convolution kernel, and the light blue area is the receptive field.

Figure 2 is a schematic diagram of 3 × 3 dilated convolutions with different dilation rates. Their receptive fields are 3 × 3 and 7 × 7, respectively. Note that a dilated convolution with a dilation rate of one is equivalent to a normal convolution. Compared with the simple stacked ordinary convolution, the dilated convolution can reduce the number of convolutional layers while obtaining a larger receptive field. Therefore, the model employs dilated convolutional blocks to extract multi-scale features of cardiac MRI.

**FIGURE 3:**
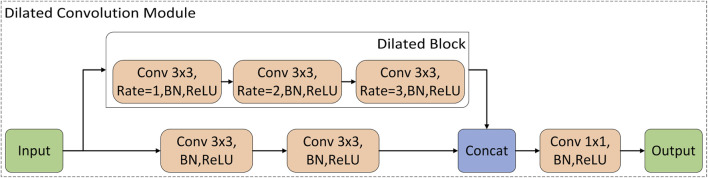
The structure of the dilated convolution module.

**FIGURE 4:**
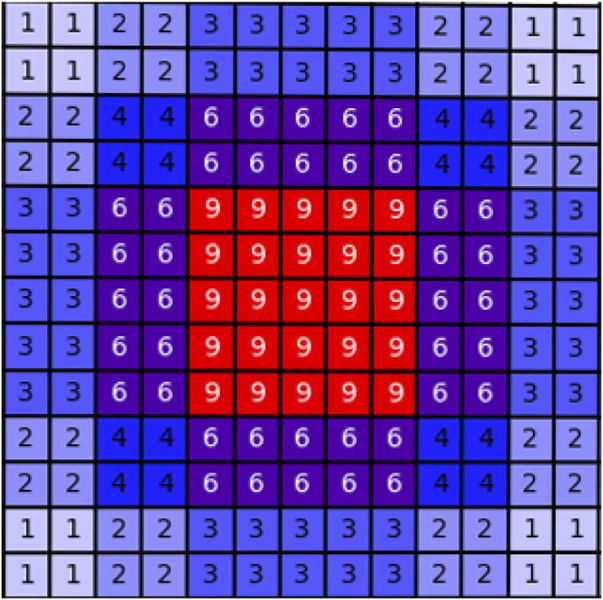
Schematic diagram of the receptive field of the dilated block. The numbers in the grid represent the number of convolutions.

The structure of the dilated convolution module is shown in Figure 3, which uses 3 × 3 convolution kernels with different dilation rates for multi-scale feature extraction, and forms a parallel structure with the double convolution layer of the original U-Net. The purpose of the dilated convolution is to extract the multi-scale features of the image. We set the rates to 1, 2, and 3, respectively. The resulting multi-scale features are then concatenated and then passed through a 1 × 1 convolutional layer for feature fusion. Compared with traditional convolutional layers, dilated convolutions can use fewer parameters to obtain a larger receptive field. This is very beneficial for data-limited cardiac MRI segmentation tasks. The receptive field of the Dilated block in Figure 3 is shown in Figure 4. The numbers in the grid represent the number of convolutions.

As shown in Figure 1, the overall U-Net infrastructure is adopted. We replace all double-layer 3 × 3 convolutions in the encoder and decoder with dilated convolution blocks to extract and fuse multi-scale features. And in the decoder, we upsample the features produced by each layer to the original image size, then concatenate them and perform feature fusion through a 1 × 1 convolution. The feature fusion layer does not change the size of the input features, but takes the concatenated features as the input of the 1 × 1 convolution block to generate the fused features. After this step, the feature fusion is completed, and the number of channels changes from 512 to 64.

### 3.3 Edge fusion block

We propose an edge fusion module to effectively utilize edge features, as shown in Figure 1. First, we use the existing method Zitnick and Dollár (2014) to extract the edge map and take it as one of the inputs of DFB. Second, in the decoding stage, EFB embeds the edge features into the downsampled features of the same size as the upsampled features, and concatenates them with the upsampled features. The DFB is a two-step process. First, the edge map passes through four convolution layers of size 3 × 3 to generate conditional features. Second, in order to make better use of the edge features, EFB outputs two independent branch features (*γ*, *β*) based on the conditional features. We use (*γ*, *β*) to transform the feature *X*_*ec*_ in the encoding stage into a feature *X*_*es*_ with edge sensing capability as follows:

$$EFB\left(X_{es}|\gamma ,\beta \right)=X_{ec}⊙\gamma +\beta ,$$ (2)

where ⊙ and + represent the element-wise product operation and the element-wise addition operation, respectively. The EFB performs spatial transformations as well as feature operations. As shown in Figure 1, our model uses four EFBs to integrate edge features.

### 3.4 Directional field module

We use a direction field module composed of 1 × 1 convolution to learn the direction field. Its input is the final output feature of the model decoder, and the output is the direction field with channel number of two. The background pixel of the direction field is (0, 0), which is defined as follows:

$$F\left(a\right)=\left\{\begin{array}{cc} \frac{\vec{ba}}{\left|\vec{ba}\right|}\; & a\in \text{ foreground }\\ \left(0,0\right)\; & \text{ otherwise. } \end{array}\right.,$$ (3)

where *a* represents the foreground pixel, *b* represents the pixel where *a* is located closest to the border of the cardiac tissue, and $\vec{ba}$ is the direction vector between *b* and *a*, which we normalize by distance.

The direction field module provides a direction vector for each pixel to point to the central region, which predicts the relationship between pixels. After generating the direction field, the model uses the generated direction field $F\in R^{2\times H\times W}$ to improve the output feature $M^{0}\in R^{C\times H\times W}$ to obtain the improved feature $M^{N}\in R^{C\times H\times W}$ . The features in the central region are error corrected for $M^{0}\in R^{C\times H\times W}$ , and each pixel is updated iteratively. The operation is defined as follows:

$$\forall a\in \Omega ,M^{k}\left(p\right)=M^{\left(k-1\right)}\left(a_{x}+F{\left(a\right)}_{x},a_{y}+F{\left(a\right)}_{y}\right),$$ (4)

where Ω is the image domain, *k* represents the *k*th step, *N* is the total number of iterations, and *p*_*x*_ and *p*_*y*_ represent the *x* and *y* coordinates of pixel *a*, respectively. Subsequently, $M^{N}\in R^{C\times H\times W}$ and $M^{0}\in R^{C\times H\times W}$ are concatenated as the input of the final classifier to generate segmentation results.

### 3.5 Loss function

The loss function involved in this method includes the segmentation $L_{CE}^{i}$ with U-Net as the architecture, the segmentation $L_{CE}^{f}$ after the direction field, and the direction field module *L*_*F*_. The segmentation model based on U-Net uses cross-entropy *L*_*CE*_ as the segmentation loss. *L*_*CE*_ is defined as follows

$$L_{CE}=-\underset{i}{\sum }p_{i}\log_{2}\left(q_{i}\right),$$ (5)

where *p*_*i*_ is the ground truth and *q*_*i*_ is the predicted value. Then the model selects *L*_2_-norm distance and angle distance as the loss for direction field learning

$$\begin{array}{ll} L_{F} & =\underset{a\in \Omega }{\sum }w\left(a\right)\left(\|F\left(a\right)-\hat{F}\left(a\right){\|}_{2}\right.\\ & \;\left.+\alpha \times {\left\|\cos^{-1}\left\langle F\left(a\right),\hat{F}\left(a\right)\right\rangle \right\|}^{2}\right), \end{array}$$ (6)

where *F* and $\hat{F}$ are the ground truth and the corresponding predicted direction field respectively. The hyperparameter *α* is set to one to balance L2-norm distance and angular distance. The weight on pixel *a* is represented by *w*(*a*), which is defined as

$$w\left(a\right)=\left\{\begin{array}{cc} \frac{\sum_{i=1}^{N_{cls}}\left|C_{i}\right|}{N_{cls}\cdot \left|C_{i}\right|}\; & a\in C_{i}\\ 1\; & \text{ otherwise } \end{array}\right.,$$ (7)

where $\left|C_{i}\right|$ is the total number of pixels with label *i*, and *N*_*cls*_ is the number of classes. The total loss *L*_*all*_ contains *L*_*CE*_ and *L*_*F*_, where the balance factor *λ* = one

$$L_{\mathrm{all}}=L_{CE}^{i}+L_{CE}^{f}+\lambda L_{F}.$$ (8)

**FIGURE 5:**
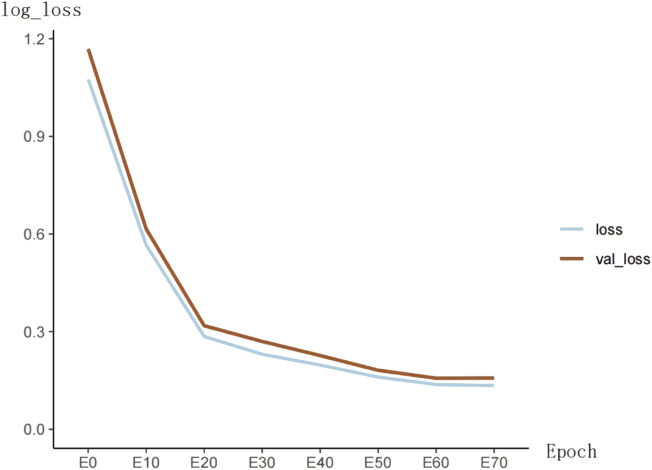
Overall structure of the proposed DDFN model.

The training loss of the model is shown in Figure 5. The loss function value decreases significantly in the first 20 epochs and then becomes slow. At the 60th epoch, the model’s loss cannot continue to decrease.

## 4 Experiment and analysis

In this section, we describe the processing of the dataset and the experimental environment. Then, we conduct ablation experiments to demonstrate the effectiveness of the model and analyze it. Finally, we compare our method with other methods on ACDC and MyoPS datasets.

### 4.1 Datasets

In this section, we introduce three different datasets: ACDC, MS-CMRSeg, and MyoPS. The datasets are all derived from challenges, and all data labels are done by experts in the relevant fields.

*ACDC 2017:* The ACDC dataset Bernard et al. (2018) contains 100 training images. These data included groups for normal cases, heart failure with infarction, dilated cardiomyopathy, hypertrophic cardiomyopathy, and right ventricular abnormalities. The dataset provides LV, RV, and MYO labels.

*MS-CMRSeg 2019:* Multi-sequence cardiac mr segmentation (MS-CMRSeg) Zhuang (2016); Zhuang (2018) dataset contains data of 45 cases. This dataset provides cardiac MRI images with three different sequences: bSSFP, LGE and T2. The sFFPS MRI is an equilibrium steady state free precession sequence. The LGE MRI is a T1-weighted gradient echo sequence. The T2 MRI is a T2-weighted, black blood spectral presaturation attenuated inversion-recovery (SPAIR) sequence. The dataset provides LV, RV, and MYO labels.

*MyoPS 2020:* The myocardial pathological segmentation (MyoPS) Zhuang (2016); Zhuang (2018) dataset provides 25 labelled MRI data. MyoPS is similar to the MS-CMRSeg dataset in that it provides cardiac MRI images with three different sequences. This dataset includes left ventricular blood pool, left ventricular blood pool, left ventricular normal myocardium, left ventricular myocardial edema, and left ventricular myocardial scar.

We use the ACDC dataset as the model training dataset and part of it as the test set. Due to the similarity and small size of MS-CMRSeg and MyoPS datasets, we take MS-CMRSeg as the training set and MyoPS as the test set. Since only LV, RV, and Myo were labeled in the MS-CMRSeg dataset, myocardial scarring and myocardial edema in the MyoPS dataset were included in the MYO classification. For the ACDC dataset, we use one-fifth of the training images as validation images and perform experiments with 5-fold cross-validation. In the validation set, we use the dice coefficient and hausdorff distance (HD) to evaluate the model. The formula of the Dice and HD evaluation index is as follows

$$Dice=\frac{2\left|R\cap R_{G}\right|}{\left|R\right|+\left|R_{G}\right|},$$ (9)

where, *R*_*G*_ represents the ground truth and *R* represents the segmentation result. The formula of the HD evaluation index is as follows

$$\begin{array}{cc} HD & =MAX\left(MAX_{X\subset O_{R}}MIN_{X\subset O_{G}}d\left(x,y\right)\right.,\\ & \;\;\;\;\left.MAX_{X\subset O_{G}}MIN_{X\subset O_{R}}d\left(x,y\right)\right), \end{array}$$ (10)

where, *O*_*R*_ and *O*_*G*_ represent the contour of segmentation result and ground truth respectively, and *d* represents the Euclidean distance between two points.

### 4.2 Implementation details

The thickness of slices in MRI is large, which easily leads to insufficient connectivity information between slices Jang et al. (2017). Therefore, the cardiac MRI was first converted into a two-dimensional image through slices. Then, in order to make better use of the batch processing mechanism, all images with a width and height greater than 256 are cropped to 256 × 256. For images less than this size, we fill them with the minimum gray value of each image.

The proposed model is trained on Nvidia RTX3090Ti GPU. We adopt Adam optimizer Kingma and Ba (2014) to assist training, and the initial learning rate is set to 10^−4^. We set up an early stop mechanism. Within 15 epochs, the evaluation dice index on the validation set does not increase by more than 0.1%, then the training is stopped, and the best model on the validation set is saved. HD can assess the difference between two sets of points. The smaller the HD value, the better the effect of the model.

The hyperparameter Settings of the model are shown in Table 1. Where, max_epoch represents the maximum number of training epochs, and early_stop_epoch represents the stop of training when loss does not decrease during continuous training for 15 epochs.

**TABLE 1:** Hyper-parameter setting of the model.

Hyper-parameter	—
Input size	256
Batch_size	8
Max_epoch	300
Early_stop_epoch	15
Initial learning rate	0.0001
Decay of learning rate	0.00001

### 4.3 The overall performance of the proposed method

**FIGURE 6:**
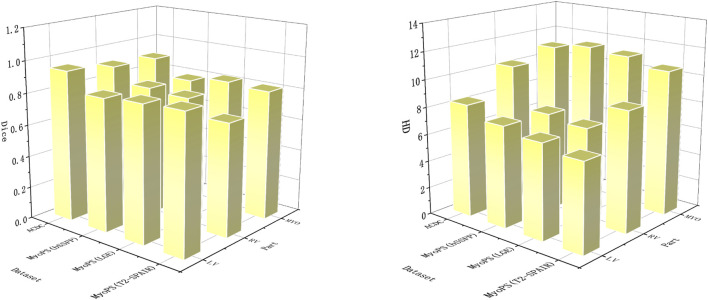
In order to obtain better visual effects, the segmentation parts and evaluation indexes are displayed in the form of three-dimensional bar charts. Dice’s score is on the left and HD’s score is on the right.

Table 2 and Figure 6 shows the performance of the proposed cardiac MRI segmentation algorithm on the ACDC and MyoPS datasets. As shown in Table 2, the average dice index and average HD index of LV, RV and MYO all reach a relatively good standard. In the ACDC dataset, the dice index of LV reaches 0.947, showing good a performance of the model. For the three different parts of the heart, the LV segmentation accuracy is the highest, while the MYO segmentation accuracy is lower. This is due to the presence of some diseases (such as myocardial infarction) in MYO, which cause changes in its appearance, which in turn increases the difficulty of segmentation. However, our proposed method still achieves a decent accuracy. The MYO value in the average HD index is larger, and it is speculated that the segmentation difficulty was increased due to the low contrast of cardiac MRI and the large change in MYO size. For the MyoPS dataset, the segmentation results are different due to the different intensity distributions of three different sequences of MRI. The intensity distribution of LGE sequence images is similar to that of bSSFP sequence images, so the variation trend of experimental results is the same. Among the segmentation results of these two sequences, the LV segmentation task achieved the highest Dice score and the lowest HD score. Among the segmentation results of T2-SPAIR sequence, LV segmentation results obtained the highest Dice score, but RV was relatively low.

**TABLE 2:** Overall performance of the proposed method.

Dataset	Dice				HD			
	LV	RV	MYO	Mean	LV	RV	MYO	Mean
ACDC	0.947	0.908	0.899	0.918	8.314	10.281	11.014	9.892
MyoPS(bSSFP)	0.830	0.818	0.794	0.814	7.456	7.299	11.481	8.745
MyoPS(LGE)	0.849	0.803	0.831	0.827	6.991	6.915	11.248	8.384
MyoPS(T2-SPAIR)	0.861	0.708	0.817	0.793	6.501	8.824	10.672	8.665

### 4.4 Network structure analysis

In this section, we perform ablation experiments on the proposed model for detailed analysis. Our model design is based on U-Net, which is a popular network for medical image segmentation tasks. Therefore, in the ablation experiments, we use U-Net as the baseline comparison model.

#### 4.4.1 Study on the dilated convolution module

The proposed model adopts a dilated convolution module to expand the receptive field and obtain multi-scale feature information. To demonstrate the effectiveness of the dilated convolution block, we change the module to the U-Net initial double convolution module and keep other configurations unchanged. It is then compared with the original model. Table 3 shows the comparison results on the ACDC dataset. As can be seen from the table, the performance of the model after removing the dilated convolution block is significantly degraded. Dice’s mean decreased from 0.918 to 0.900, while HD’s mean increased from 9.892 to 10.494. This is because the dilated convolution module can effectively expand the receptive field and extract multi-scale feature information.

**TABLE 3:** Dice/HD of our methods on ACDC dataset.

Methods		Dice				HD			
		LV	MYO	RV	Mean	LV	MYO	RV	Mean
DDFN(Ours)	—	0.947	0.899	0.908	0.918	8.314	11.014	10.281	9.892
DDFN	w/o dilated convolution module	0.932	0.881	0.889	0.900	8.914	11.881	10.712	10.494
DDFN	w/o multi-scale feature fusion	0.940	0.891	0.899	0.910	8.901	11.323	10.587	10.270
DDFN	w/o direction field module	0.939	0.886	0.896	0.905	9.257	12.104	11.951	11.104
DDFN	w/o edge fusion block	0.938	0.885	0.893	0.904	8.502	11.357	10.417	10.092

In addition, we also conduct ablation experiments for the effect of different dilated rates on the experimental results. In the dilated convolution module, we set the dilated rate to three groups of {1, 2, 3}, {1, 2, 5}, {1, 3, 5} respectively for comparison. The experimental results are shown in Table 4. The results show that the model performs the best when the dilated rate is set to (1, 2, 3). Therefore, we apply this setting to our model.

**TABLE 4:** Ablation experiments with different dilation rate settings.

Settings	Dice			
	LV	MYO	RV	Mean
(1, 2, 3)	0.947	0.899	0.908	0.918
(1, 2, 5)	0.942	0.883	0.890	0.905
(1, 3, 5)	0.939	0.890	0.889	0.906

#### 4.4.2 Study on the multi-scale fusion module

To demonstrate the effectiveness of the multi-scale fusion module, we remove the entire multi-scale module and keep other processing steps unchanged. The experimental results on the ACDC dataset are shown in Table 3. The Dice and HD values of the model using multi-scale modules have been improved. Therefore, the experimental results can prove that multi-scale fusion module is beneficial to cardiac MRI segmentation task. This is because the multi-scale fusion module can fully utilize the features of each layer of the decoder.

#### 4.4.3 Study on the edge fusion block

The role of the EFB is to use edge features for more accurate segmentation of MRI. To demonstrate the effectiveness of the EFB, we performed an ablation experiment on the EFB. The ablation results of EFB are shown in Table 3. We deleted the EFB and kept the other procedures unchanged for comparison. As can be seen from the table, both Dice and HD values have been improved. The results show that the module effectively uses edge features, which is conducive to cardiac MRI segmentation.

#### 4.4.4 Study on the direction field module

Our method utilizes the direction field module to learn a direction field, which represents the direction relationship between each pixel. Its function is to improve the segmentation feature map. To demonstrate the effectiveness of this module, we analyze the impact of the direction field module on the segmentation task. In ablation experiments, we remove the direction field module of DFFN and keep other settings unchanged. It can be seen from Table 2 that the precision of the model decreases significantly after the direction field module is removed. In particular, the average of HD increased from 9.892 to 11.104. This proves that the direction field module can effectively improve the output features and obtain better cardiac segmentation results.

### 4.5 Comparison with existing methods

In this section, the proposed cardiac MRI segmentation method is compared with other mainstream networks. Including U-Net Ronneberger et al. (2015), U-Net++ Zhou et al. (2018), DeeplabV3+ Chen et al. (2018), Segnet Badrinarayanan et al. (2017), Distance Map Regularized (DMR) Dangi et al. (2019) and SK-Unet Wang et al. (2021). The above methods are encoder - decoder structure. U-Net is a very classical model in medical image segmentation, while Segnet is one of the earliest multi-pixel segmentation models. DeeplabV3+ is a conventional semantic segmentation method and has achieved very good results in VOC2012 dataset. U-Net ++ is an improvement on the basis of U-Net, which alleviates the unknown network depth through effective integration of features of different depths. CE-Net integrates dense convolution and residual structure into the model to improve the segmentation performance. DMR is a distance graph regularized image segmentation model. SK-Unet utilizes the selection kernel module and residual module to improve the U-Net model. This section compares the above methods with our proposed ones. To be fair, the parameter settings are all the same as the proposed method.

#### 4.5.1 Experiments on ACDC dataset

Table 5 shows the comparison results of all methods on the ACDC dataset. Experimental results show that compared with other methods, our proposed method has certain advantages and dice value has been significantly improved. Among them, DeeplabV3+ performs poorly, and it can be seen that it is not suitable for medical image segmentation. As a baseline model, U-Net has better performance, but there is still room for improvement. U-Net++ has achieved obvious results after improving U-Net, and the Dice value has increased from 0.912 to 0.928. DMR and SK-Unet are very effective as recent cardiac segmentation methods. Compared with these methods, the average dice value and average HD value of our method reached 0.918 and 9.892. Among them, the dice value of LV reached 0.947, the RV reached 0.908, and the segmentation of MYO is difficult due to heart disease, which is 0.899. Overall, our method achieves competitive results for segmentation of various parts of the heart. This is because our model can effectively extract and utilize multi-scale information without causing the loss of feature information or the increase of useless information. In addition, the model retains the edge information to make the results more accurate.

**TABLE 5:** Dice and HD of different segmentation models on ACDC dataset are compared quantitatively.

		U-net	DeeplabV3+	Segnet	U-net++	DMR	SK-unet	Ours
Dice	LV	0.912	0.824	0.919	0.928	0.929	0.932	**0.947**
	RV	0.857	0.711	0.861	0.874	0.884	0.882	**0.908**
	MYO	0.813	0.756	0.823	0.842	0.853	0.873	**0.899**
—	Mean	0.861	0.764	0.868	0.881	0.888	0.895	**0.918**
HD	LV	9.318	19.554	9.813	9.051	9.248	8.898	**8.314**
	RV	11.899	24.158	12.015	11.459	11.548	10.945	**10.281**
	MYO	14.176	27.456	14.991	13.546	14.354	12.458	**11.014**
—	Mean	11.797	23.722	12.273	11.352	11.716	10.767	**9.892**

The values in bold are the best results.

**FIGURE 7:**
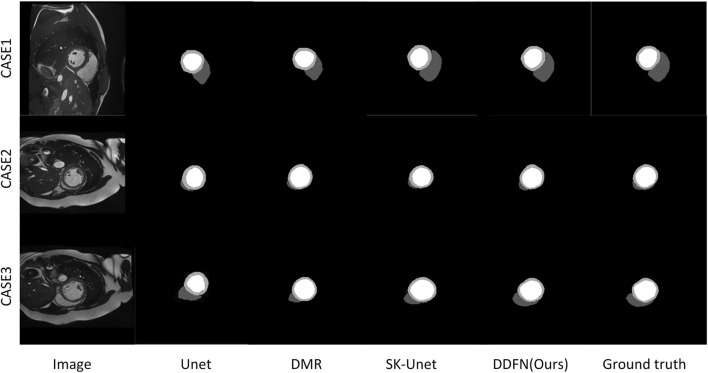
The variation of loss.

Figure 7 presents a visual comparison of the proposed cardiac MRI segmentation method against other methods. We select the segmentation results of three different slices for comparative display. Among them, U-Net can accurately segment LV parts, but cannot segment RV and MYO well. The remaining other models can segment the three parts of the heart well, but there are still some shortcomings. The segmentation results of DMR are prone to omissions, and SK-Unet is prone to over-segmentation. Our segmentation result is the closest to ground truth. However, for some very fine edge structures, our method still falls short. With the deepening of the layer number of convolutional network, the edge information is easy to be gradually blurred. Briefly, the deep convoluted layer cannot obtain better boundary information. Therefore, fine edges are not easy to recover. These fine edge structures are difficult to segment manually even for experienced experts.

#### 4.5.2 Experiments on MyoPS dataset

Since the MyoPS dataset contains MRI with three different sequences: bSSFP, LGE, and T2-SPAIR, we designed three sets of comparative experiments to verify the effectiveness of the model.

##### 4.5.2.1 Comparison of results on bSSFP sequence MRI

Table 6 shows the experimental comparison results of our method and other methods on bSSFP sequence images. It can be seen that compared with the classical U-Net method, our method improves the RV segmentation accuracy by 2.11%. The segmentation accuracy was also improved in MYO and LV segmentation tasks. And compared with other methods, our method can segment more accurately.

**TABLE 6:** Dice and HD of different segmentation models on MyoPS dataset are compared quantitatively.

bSSFP MRI								
—	—	U-net	DeeplabV3+	Segnet	U-net++	DMR	SK-unet	Ours
Dice	LV	0.812	0.734	0.821	0.820	0.822	0.825	**0.830**
	RV	0.797	0.691	0.792	0.809	0.811	0.814	**0.818**
	MYO	0.783	0.695	0.806	0.789	0.790	0.791	**0.794**
HD	LV	7.618	12.798	7.583	7.499	7.491	7.477	**7.456**
	RV	7.576	13.186	7.491	7.545	7.557	7.348	**7.299**
	MYO	11.971	20.854	11.815	11.713	11.706	11.648	**11.481**
LGE MRI								
—	—	U-Net	DeeplabV3+	Segnet	U-Net++	DMR	SK-Unet	Ours
Dice	LV	0.837	0.731	0.844	0.842	0.834	0.840	**0.849**
	RV	0.764	0.679	0.772	0.778	0.769	0.792	**0.803**
	MYO	0.807	0.697	0.818	0.831	0.827	0.829	**0.831**
HD	LV	7.215	12.948	7.158	7.115	7.128	7.112	**6.991**
	RV	7.954	13.485	7.758	7.147	7.168	7.135	**6.915**
	MYO	12.015	19.942	11.849	11.428	11.489	11.408	**11.248**
T2-SPAIR MRI								
—	—	U-Net	DeeplabV3+	Segnet	U-Net++	DMR	SK-Unet	Ours
Dice	LV	0.848	0.795	0.850	0.853	0.852	0.857	**0.861**
	RV	0.684	0.624	0.683	0.689	0.691	0.695	**0.698**
	MYO	0.795	0.742	0.792	0.798	0.801	0.811	**0.817**
HD	LV	6.518	10.548	6.517	6.521	6.517	6.510	**6.501**
	RV	8.849	15.984	8.850	8.853	8.842	8.834	**8.824**
	MYO	10.742	19.571	10.743	10.738	10.698	10.691	**10.672**

The values in bold are the best results.

##### 4.5.2.2 Comparison of results on LGE sequence MRI

The comparison results are shown in Table 6. The intensity distribution of MRI of LGE sequence is similar to that of bSSFP sequence, so the trend of MRI segmentation accuracy of the two sequences is similar. Our method outperforms other methods on cardiac MRI segmentation tasks. In addition, the proposed method achieves the highest Dice score on LV, RV and MYO segmentation, and the lowest Hausdorff distance score.

##### 4.5.2.3 Comparison of results on T2-SPAIR sequence MRI

The intensity distribution of T2-SPAIR MRI was different from that of the previous two sequences. Table 6 shows the experimental results. It can be seen that all segmentation methods perform poorly when segmenting RV. When segmenting lv, the segmentation accuracy of the proposed method is slightly higher than that on the other two sequences. For the MYO site, the proposed method performed well on all three sequences of MRI. Similarly, in the MRI segmentation task of T2-SPAIR sequence, our proposed method performs well.

## 5 Conclusion

This paper proposes a cardiac MRI segmentation method utilizing multi-scale features and orientation field modules. This method makes full use of multi-scale features, and effectively improves the output features through the directional field module, thereby obtaining better segmentation accuracy. In addition, the model also uses edge features to further improve the segmentation performance. Our limitation is that with the deepening of the convolution layer, some small details are easily lost and cannot be recovered. In the future work, we will try to provide global context information for all the convolutional layers in the decoder to preserve the more easily ignored details.

## Data Availability

Publicly available datasets were analyzed in this study. This data can be found here: https://www.creatis.insa-lyon.fr/Challenge/acdc/index.html.
